# Pigment Dispersion Syndrome Progression to Pigmentary Glaucoma in a Latin American Population

**DOI:** 10.5005/jp-journals-10008-1187

**Published:** 2016-02-02

**Authors:** Hector Fernando Gomez Goyeneche, Diana Patricia Hernandez-Mendieta, Diego Andres Rodriguez, Ana Irene Sepulveda, Jose Daniel Toledo

**Affiliations:** Senior Consultant and Head, Department of Ophthalmology, Hospital Militar Central Universidad Militar Nueva Granada, Bogotá, Colombia; Ophthalmologist, Department of Ophthalmology, Hospital Militar Central Universidad Militar Nueva Granada, Bogotá, Colombia; Ophthalmologist, Department of Ophthalmology, Hospital Militar Central Universidad Militar Nueva Granada, Bogotá, Colombia; Ophthalmologist, Department of Ophthalmology, Hospital Militar Central Universidad Militar Nueva Granada, Bogotá, Colombia; Ophthalmologist, Universidad Militar Nueva Granada, Bogotá, Colombia

**Keywords:** Intraocular pressure, Pigmentary glaucoma, Pigment dispersion syndrome, Progression.

## Abstract

**Objective:** To determine the progression of pigment dispersion syndrome (PDS) into pigmentary glaucoma (PG) in a population at the Central Military Hospital in Bogotá, Colombia.

**Materials and methods:** A retrospective study was conducted, based on a review of medical records of patients with PDS evaluated in the Glaucoma Clinic. Data were collected in a database in excel and subsequently analyzed with the software Statistical Package for the Social Sciences (SPSS), performing Chi-square test analysis and Spearman’s rho test.

**Results:** Forty-eight eyes of 24 patients were included. Forty-two percent were women and 58% were men. Pigmentation of the trabecular meshwork was the most frequent clinical sign (100%), followed by Krukenberg’s spindle (91.7%), the least frequent were the iris concavity and iris heterochromia (4.2%), the average of the spherical equivalent was of - 1.33 (SD 2.07).

The rate of conversion of PDS to PG was 37.5%, after an average follow-up of 50.7 months. Having an intraocular pressure (IOP) greater than 21 mm Hg was statistically the only significant risk factor for conversion.

**Conclusion:** We found several differences in frequency and clinical signs in these patients in contrast to previous data, probably due to different racial characteristics.

The rate of progression is similar to previous reports despite of heterogeneity of these. Having IOP > 21 mm Hg was the only risk factor associated with progression in this sample.

**How to cite this article:** Gomez Goyeneche HF, Hernandez-Mendieta DP, Rodriguez DA, Sepulveda AI, Toledo JD. Pigment Dispersion Syndrome Progression to Pigmentary Glaucoma in a Latin American Population. J Curr Glaucoma Pract 2015;9(3):69-72.

## INTRODUCTION

The pigment dispersion syndrome (PDS) and pigmentary glaucoma (PG) are two consecutive and progressive stages of an uncommon pathological process, characterized by a release and subsequent deposit, especially in anterior chamber and trabecular meshwork^1,2^ of pigment granules originated in the posterior pigment epithelium of iris, these situation may result in increased intraocular pressure (IOP) and optic nerve damage.^3,4^

The exact triggers for this condition are not fully understood, there are many factors involved including genetic,^5^ immune-mediated damage,^6^ among others. John et al^7^ and Chang et al,^8^ described mutations in TYRP 1 and GPNMB genes in animal models as DBA/2J (D2) mice that induced iris depigmentation, pigment dispersion and optic nerve damage.

Through observational studies and high resolution ultrasonography, several authors had proposed a pathophysiological mechanism called reverse pupillary block which states that a transient increase in pressure occurs in the anterior chamber compared to the posterior chamber, generating a posterior arc of iris stroma leading a touch between the posterior pigment epithelium of iris and ciliary zonule, which leads to pigment dispersion.^9-13^

Pigment dispersion syndrome and PG are not common for Latin American population, and there is no previous information in databases about the behavior and evolution of these conditions. To our knowledge, this is the first study of this group describing the rate of progression from pigmentary dispersion syndrome to PG.

## MATERIALS AND METHODS

We conducted a descriptive, retrospective study with patients referred with PDS and PG to the glaucoma service of the Central Military Hospital (referral center) and to the Glaucoma Department in an outpatient service of a private clinic in Bogotá (Colombia).

The patients were selected and classified for PDS according to the inclusion criteria, PDS was: Shaped radial slitting defects in the midperipheral iris transillumination, Krukenberg’s spindle or increased pigmentation of the trabecular meshwork or other intraocular structures.

All the patients with pigmentary glaucoma―any of the previous signs plus glaucomatous optic nerve damage, increased cupping or abnormal disk appearance, and glaucomatous visual field defects were excluded.

Patients with ocular comorbidities (that also cause pigment dispersion in the anterior segment) were excluded: pseudoexfoliation syndrome, history of uveitis, uveitic glaucoma, pseudophakia, ocular trauma or any other causes of inflammatory disease (infectious, autoimmune).

Medical records from 2007 to July 2014 were reviewed, including patients diagnosed with PDS. A database was constructed including clinical and demographic characteristics. Forty-eight eyes of 24 patients fulfilled the inclusion criteria.

The variables were analyzed by the Statistical Package for the Social Sciences (SPSS) tool (SPSS Inc., Chicago, IL, USA) for statistical analysis. Chi-square test and Spearman’s rho test were conducted to determine key variables for progression of PDS to PG.

## RESULTS

**Graph 1:**
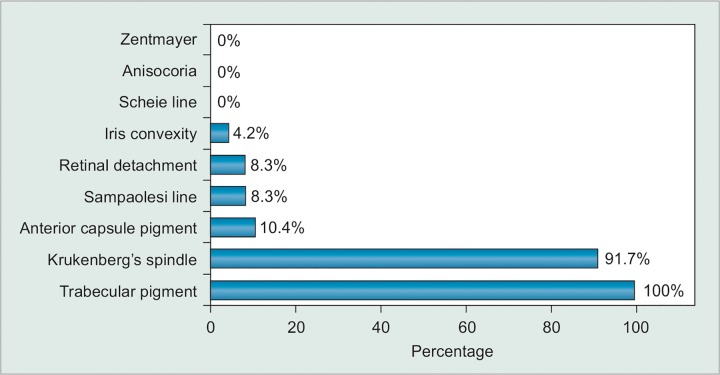
Clinical findings

During evaluation of the clinical signs, we found that pigmentation of the trabecular meshwork was the most frequent sign (100%), followed by Krukenberg’s spindle (91.7%), which in most of the cases were not fully manifested, but just faint endothelium pigment. Transillumination defects were uncommon (8.3%). Other clinical features can be seen in (Graph 1).

Patients were mostly men (58 %) *vs* women (42%) and the average age at initial visit was 53.2 years (SD 12.9).

Thirty-two eyes (66.6%) were myopic, with global average of spherical equivalent of - 1.33 D (SD 2.07) and the initial IOP was 19.2 mm Hg (SD 3.8). Only 25% of patients had a history of glaucoma in a first degree relative. Other demographic data and features at first visit can be seen in Table 1.

**Table 1:** **Table 1:** Clinical and demographic data of the patients with PDS

*Clinical features*		*Male (n = 14)*		*Female (n = 10)*	
Number of eyes		28		20	
Average age initial visit		56.9 (23-67)		50.6 (41-76)	
Mean spherical equivalent		–1.69 D ± 1.8		–0.84 D ± 2	
Family history of glaucoma		21.4%		30%	
IOP mean initial visit		18.9		17.6	

PDS: Pigment dispersion syndrome; IOP: Intraocular pressure

**Table 2:** **Table 2:** Odds ratio and p-value

*Characteristics*		*Odds ratio*		*p-value*	
Male		0.76		0.49	
Age > 50 years		0.82		0.59	
Familiar history of glaucoma		0.30		0.24	
Intraocular pressure > IOP 21 mm Hg		0.002		0.003	

Average follow-up was 50.7 months. In the final evaluation, 30 eyes had PDS and 18 eyes had PG, with a conversion rate of 37.5% (35.7% males and 40% females) based on visual fields and optic disk changes.

During follow-up, 41% of eyes required surgical intervention, management included argon laser trabeculo-plasty (ALT) 19%, selective laser trabeculoplasty (SLT) 17.5% and trabeculectomy 3.5%.

We analyzed initial characteristics of these patients to determine risk factors for progression, only IOP > 21 mm Hg was statistically significant for progression of PDS to PG (p = 0.003) (OR 0.002). None of the other factors like age, gender, degree of myopia, and family history of glaucoma had significant value (Table 2).

## DISCUSSION

Pigment dispersion syndrome and PG are rare and often misdiagnosed entities. Since initial descriptions made by sugar,^14^ there were many papers describing its characteristics, demography and rates of progression.^2,4,15,16^

To our knowledge, this is the first study to analyze the demographics, clinical signs and behavior over time in a Latin American population.

The average age at the time of first visit was 53.2 years (SD 12.9), in contrast to other studies^15,16^ were these findings are between 30 and 40 years, this could be explained for two reasons: first, a low rate of suspicion in previous ophthalmology consultations, due to subtle clinical findings, which delays appropriate recognition and follow-up; second, the patients in this study are third and fourth levels referral, and often their derivation to a glaucoma specialist is due to high cup/disk ratio, ocular hypertension or even glaucomatous damage.

The most common clinical finding was trabecular meshwork pigmentation, followed by Krukenberg’s spindle, these last finding was often very subtle and required gonioscopic examination, which is not routinely performed in general ophthalmology practice.

Defects in iris transillumination are not common in our sample as well as in other noncaucasian patients^17^ probably due to low incidence of light color iris compared to the dark and morphological compact iris stroma of Latin population. These findings suggest that the clinical presentation in our population can be different, and highlight the importance of a careful and complete clinical exam, including gonioscopy to enhance rate of diagnosis.

Regarding other clinical features in this sample, most of them were myopic with a mean spherical equivalent of - 1.66 D (SD 2.07) which is consistent with previous studies^9,15,16^ but with a lower degree of myopia compared with these works, maybe due to lesser prevalence of this defect in our population compared with others.

In this study, we found a rate of progression of PDS to PG of 37.5% during evaluation of 4.2 years average. However, we have to point out several limitations, related to small number of patients, short and heterogeneous follow-up period and selection bias, because these patients were referred for specialized glaucoma assessment and probably with high-risk conditions for progression to PG.

There are several studies in the literature with heterogeneous results of rates of progression. Richter et al found a progression rate of 18% at 3.7 years, Farrar et al^2^ conducted a study with 18 patients, with a maximum follow-up of 14 years, in which they found a progression rate of 50%, Migliazzo et al^15^ included 37 patients, followed for 35 years, with a progression of 35%. Siddiqui et al^16^ included 11 patients with postoperative results of 15 years, with a progression rate of 15%. These data have been summarized in Table 3.

We analyze different variables recognized as possible risk factors^1,9^, such as age, gender, degree of myopia, family history of glaucoma and IOP, and only the last was statistically significant as a risk factor for progression in this sample (OR 0.002, p = 0.003) which is concordant with other studies, such as Siddiqui et al;^16^ however, we consider limitations again related to a selection bias due to choice and selected population referred for glaucoma assessment.

**Table 3:** **Table 3:** Reports of conversion rates from PDS to PG

*Trial*		*N*		*Average* * follow-up* * (io years)*		*Progression* * rate*	
Richter et al^3^		32		2.3		18%	
Farrar et al^2^		18		4.3		50%	
Migliazzo et al^15^		37		17.2		35%	
Siddiqui et al^16^		11		8		15%	
Actual trial		31		4.2		37.5%	

PDS: Pigment dispersion syndrome; PG: Pigmentary glaucoma

Although this study has several limitations, we consider it has an important approach to the behavior of these conditions in Colombian population. There are many questions that remain inconclusive, like the real benefit in this group of patients of procedures, such as laser peripheral iridotomy (LPI); Karickhoff suggested that LPI may be a potential treatment for PG.^11^ Different studies suggest that there was no benefit of LPI in preventing progression from PDS with ocular hypertension to PGA,^18,19^ but LPI, performed in high-risk eyes, reduced the rate of IOP elevation to the same level as the low-risk eyes.^20^

There are many questions that remain unanswered like role of ALT or SLT, long-term benefit of medical and surgical managements in terms of reducing rates of progression, issues on which medical literature does not completely agree.^18,20^ More investigation is required in this population,^21^ because, as we showed it may have different features compared with classical description in Caucasians and the traditional signs including iris transillumination defects, pronounced corneal endo-thelial pigmentation, posterior iris bowling and visible anterior iris stromal pigment dusting are absent in most cases. Ultrabiomicroscopy and anterior segment optical coherence tomography (AS-OCT) studies could be helpful to answer these questions.
